# Global changes to the chemotherapy service during the covid-19 pandemic

**DOI:** 10.1177/10781552211015767

**Published:** 2021-05-13

**Authors:** Man-Chie Chow, Pinkie Chambers, Georgina Singleton, Jignesh Patel, Silvie Cooper, Charlotte Mythen, Elysse Bautista-González, Georgia Chisnall, Nehla Djellouli, Benjamin Thwaites, Ian CK Wong, Cecilia Vindrola-Padros

**Affiliations:** 1 Pharmacy Department, University College London Hospitals NHS Foundation Trust, UCLH-UCL Centre for Medicines Optimisation Research and Education, London, UK; 2 Department for Targeted Intervention, UCL/UCLH Surgical Outcomes Research Centre, Centre for Perioperative Medicine, University College London, London, UK; 3 Health Services Research Centre, National Institute for Academic Anaesthesia, Royal College of Anaesthetists, London, UK; 4 Rapid Research Evaluation and Appraisal Lab (RREAL), London, UK; 5 Department of Anaesthesia and Perioperative Medicine, University College London Hospitals NHS Foundation Trust, London, UK; 6 Department of Applied Health Research, University College London, London, UK; 7 Institute of Epidemiology and Healthcare, University College London, London, UK; 8 UCL Institute for Global Health, London, UK

**Keywords:** Covid-19, coronavirus, chemotherapy, anti-cancer therapy, healthcare

## Abstract

**Purpose:**

In response to the COVID-19 pandemic, changes to chemotherapy services were implemented as a means of managing imposed workload strains within health services and protecting patients from contracting COVID-19. Given the rapidly evolving nature of the pandemic many changes were rapidly adopted and were not substantiated by robust evidence. This study aimed to describe the changes adopted internationally to chemotherapy services, which may be used to guide future changes to treatment delivery.

**Methods:**

A survey was developed to understand the impact of COVID-19 on the delivery of systemic anti-cancer therapies (SACT). It comprised 22 questions and examined the strategies implemented during the pandemic to prioritise and protect patients receiving SACT and the participants’ professional opinion of the strategies employed. The survey was available in English, Spanish and French and was distributed via professional bodies.

**Results:**

129 responses were obtained from healthcare professionals working across 17 different countries. 45% of institutions had to implement treatment prioritisation strategies and all hospitals implemented changes in the delivery of treatment, including: reduction in treatments (69%), using less immunosuppressive agents (50%), allowing treatment breaks (14%) and switching to oral therapies (45%). Virtual clinic visits were perceived by participants as the most effective strategy to protect patients.

**Conclusions:**

The pandemic has forced chemotherapy healthcare professionals to adopt new ways of working by reducing health interactions. Many areas of research are needed following this period, including understanding patients’ perceptions of risks to treatment, utilisation of oral treatments and the impact of treatment breaks on cancer outcomes.

## Background

Internationally, the SARS-CoV-2 pandemic has impacted the treatment of cancer patients.^1,2^ Continuity of cancer care is critical in optimising patient outcomes; however, continuing treatments as usual, particularly when infection prevalence remains high, can be challenging. Reports have highlighted that, in the course of the pandemic, there have been reduced numbers of patients diagnosed with cancer.^3^ In addition, chemotherapy services in some countries were also seeing fewer patients in the hospital setting in order to minimise healthcare interactions to reduce the transmission of COVID-19 infections in healthcare settings.^4^ Changes in models of care to accommodate the demand on healthcare systems generated by the rapid increase in COVID-19 patients also led to the cancellation of non-urgent treatments.^5^ Furthermore, the requirement for social distancing in the workplace has implications on the preparation and administration of treatments.^6^

Chemotherapy is known to be immunosuppressive, and concerns that this would result in a greater risk of suffering from severe COVID-19 symptoms may have caused patients and clinicians to temporarily suspend or change cancer treatment. At the start of the pandemic, decisions on the prioritisation of patients in the UK were guided by the National Institute of Clinical Excellence (NICE) and the main suggestion was to continue delivering therapies with curative intent.^7^

Anecdotal reports from different regions and countries showed that different strategies were being adopted to delay or contain the virus. Social distancing measures were adopted in virtually every country affected by the disease and this meant rapidly adapting to new ways of working. Examples of these were using self-administration of some treatments and more oral therapies. Understanding and sharing these measures was important, particularly by those countries affected early in the pandemic. In order to understand this variation at a global level, we developed a survey to explore the impact of COVID-19 on the delivery of systemic anti-cancer treatment (SACT).

We aimed to describe the main policies or guidelines being implemented for cancer treatment and strategies to mitigate the impact COVID-19 has had on cancer care.

## Methods

The study used on an online questionnaire designed to collect quantitative and qualitative data on: participants’ awareness of guidelines and policies concerning the prioritisation and protection of cancer patients; the strategies implemented to prioritise and protect patients receiving systemic anti-cancer therapies; and participants’ opinion of the effectiveness of these strategies. The survey contained 22 questions and was available in English, French and Spanish (the survey instrument can be found in Supplementary file 1).

### Questionnaire development

The study questionnaire was designed to be short as it was understood that participants would be busy at the time of data collection. The majority of questions were multiple-choice, and two were open-ended free-text questions. The first free-text question asked participants to describe the changes that they perceived were successful and unsuccessful and why. The free-text question allowed respondents to expand on answers in previous questions and raise concerns or issues that had not been addressed elsewhere. Supplementary questions enquiring about respondent characteristics and how they were sent the survey were also included.

### Content validity

Content validity for the questionnaire was quantified using the content validity index (CVI) as recommended by Polit et al..^8^ An expert panel was assembled, which included experts with a background in survey design, chemotherapy treatment, and the delivery of healthcare in the context of infectious epidemics. They were asked to comment on three domains as recommended by Grant & Davis:^9^ the relevance of each question; the clarity of each question; and the comprehensiveness of the entire survey. All questions were found to have a CVI >0.78, therefore not requiring question revision in line with the model described by Polit and colleagues. Revisions were made to the wording of the questions to improve clarity and understanding based on feedback from the expert panel. The panel unanimously agreed that the questionnaire was comprehensive and covered the important areas. Once the questionnaire was translated in French and Spanish, it was again validated by two clinicians to ensure the questions were interpreted correctly.

### Sampling

The survey was open between 15th May 2020 and 9th June 2020 and was disseminated via professional bodies and through snowball-sampling techniques to target healthcare professionals who had a role in delivering cancer care around the world. Professional bodies included The British Oncology Pharmacists Association, The International Society of Oncology Pharmacists, The Royal College of Radiologists, The United Kingdom Nurses Association and The British Urology Group.

### Sample size

The population for this study were healthcare professionals involved with delivering care to cancer patients. A sample size of 100 was calculated using the following formula, commonly used when population mean is unknown, where n is the sample size, Z the score for 95% confidence level, p the maximum variability of the population at 50% and e the sampling error.

n=(Z)2p(1−p)/e2

Considering Z = 1.96 for 95% confidence level, p = 0.5 and a sampling error of 10%, n = 96.125. For rounding reasons, we believed a sample of 100 participants was justified.

### Survey administration

The study, including the questionnaire, was approved by the University College London Research Ethics Committee (6862/005).

The questionnaire was administered using the Opinio Platform. Members of the societies named above were initially contacted via an email inviting participation. Societies that approved the survey also used their social media platforms to disseminate the survey. The survey introduction page acted as a consent form and participant information sheet (Supplementary file 1). It stated that consent for data being used for specified purposes was implied by participating in the survey.

### Missing data

We did not use ‘forced answering’ to avoid ‘missing data’ from our online questionnaire for the open ended questions . We allowed respondents to leave blank questions as we believed forced answering could result in inaccurate data. Missing data are presented within the results.

### Data analysis

Data were exported from Opinio and analyses were conducted using STATA 15. Results are presented as count (%).

Free-text qualitative answers were compiled in a single list and were left unedited (no corrections for spelling or grammar). Data were analysed in Excel using framework analysis^10^ to allow for the identification of patterns across the data set. A broadly descriptive type of thematic analysis was employed when developing the themes. Codes were derived from the data. A review of the coding of the dataset, including the codes used, were performed by two members of the research team to cross-check the coding and the dataset (CM, SC). A third researcher cross-checked a percentage of the coding performed on the full dataset (CVP). Themes were identified with relevant data collected under each theme and reread to ensure the themes appropriately captured the views and beliefs of respondents.

## Results

The questionnaire was sent to approximately 500 relevant clinicians (nurses, pharmacists and doctors). In total, 129 responses with participants from 17 countries were obtained. Table 1 shows that the majority of participants completed the survey in English and 46% were from the United Kingdom.

**Table 1.** Overview of participants.

**Parameter**	
Number of participants (N)	129
Language	
English	100 (78%)
Spanish	29 (22%)
Country	
UK	59 (46%)
Spain ("Espa√±a")	17 (13%)
Australia	14 (11%)
Argentina	9 (7%)
USA	5 (4%)
Hong Kong	5 (4%)
China	4 (3%)
New Zealand	3 (2%)
Canada	3 (2%)
Mexico	3 (2%)
Nigeria	2 (2%)
Ireland	1 (1%)
Zambia	1 (1%)
United Arab Emirates	1(1%)
Brunei	1 (1%)
Professional group	
Pharmacist	72(56%)
Doctor	50 (39%)
Clinical nurse specialist	1 (1%)
Chemotherapy nurse	1 (1%)
Other	5 (4%)
Institution type	
Public	111(86%)
Private	11 (9%)
Hybrid (public & private)	7 (5%)

60% participants reported some SACT had been cancelled or postponed and reasons for these are detailed in Table 2. In the majority of institutions, the reason given for this was a revised individual patient risk benefit balance in view of the COVID-19 pandemic; shortages of staff, equipment or resources to shield patients from COVID-19 were cited less frequently as reasons for treatments not continuing.

**Table 2.** Reason for treatments cancelled/postponed. Total number of participants reporting treatments were cancelled or postponed was 77.

Reason	Number stating reason (%)
Revised individual risk-benefit balance (in view of COVID-19)	74 (96%)
Lack of staff owing to redeployment to other clinical areas	4 (5%)
Lack of staff owing to staff illness or isolation	4 (5%)
Lack of bed capacity and/or equipment	5 (6%)
Insufficient resources to shield cancer patients from COVID-19	6 (8%)
Cancer care has moved to another site	2 (3%)
Other	9 (12%)
Reasons specified:	
Patient choice, type of treatment, patient fearful of visiting the hospital, hospital choice to reduce treatments and lockdown restrictions impeded patient travel	

45% of institutions had to implement prioritisation strategies for chemotherapy and this was mainly seen in the United Kingdom. Not all hospitals in the United Kingdom were prioritising treatments and some were continuing all treatments as usual. However, all participants reported the implementation of strategies to protect patients from unnecessary contacts. These measures included: reduced face-to-face interactions such as telephone clinics; the reduced frequency of non-essential follow-ups; and the use of personal protective equipment (PPE). Table 3 describes the changes to chemotherapy treatments that were reported in the survey. 69% reported changes in durations of treatment and 45% implemented the change of switching therapies from intravenous to oral in an attempt to reduce patient visits.

**Table 3.** Changes made to anti-cancer treatments.

Changes to anticancer treatments	Frequency	Percentage of participants (n = 129)
Reduced frequency or duration of treatment	89	69%
Less immunosuppressive regimens	65	50%
Increase in primary prophylaxis, e.g. antibiotics, colony stimulating factors.	65	50%
Changes in route of administration (intravenous to oral)	58	45%
Changes to treatment timing, i.e. breaks	18	14%
Other changes to anticancer therapy	18	14%
Missing values	16	12%

Participants were asked which strategies were most effective and least effective and these were answered by 60 participants. The following themes emerged in our analysis in relation to the effectiveness of these strategies.

### Virtual visits

Over 60 participants stated that reducing visits and hospital footfall was effective. Virtual (telephone and online) clinics were reported and described by one participant as “*It gives time for us to focus on a few patients at a time and offer quality clinical service. It also shrinks the chance of infection for both patients and caregiver to a minimum*”. Some institutions were only adopting this for stable patients. This method of seeing patients was not seen as ideal for all and concerns were raised about the psychological impact to patients as described by one doctor: “*Telephone consultations can work well for some patients, but patients still need to be seen from time to time to accurately assess their fitness, tolerability of treatment and to offer patients the psychological support they have when seen by their team*.'”

In addition to virtual assessments, many were using postal deliveries to supply oral chemotherapy to patients and had a positive experience of this service. However, one participant referred to posting medicines as “*ineffective and reported failure of treatments to arrive on time*”.

### COVID-19 screening prior to hospital visits and PPE

20 participants reported both staff and patient screening was occurring at the time of this survey. Screening of patients was carried out through phoning and ensuring a patient had no COVID-19 symptoms and also through swab testing, as described by one respondent: “*Phone screening of each patient before arrival into clinic. We have picked up patients who needed to be referred for COVID testing and also who had travel history which meant we needed to delay treatment*.” PPE was thought of as essential to treat patients safely and reduce transmission.

### Risk stratification/tailored treatment/prioritisation

15 respondents commented on treatment breaks and switches to oral therapies from intravenous to keep patients away from hospital where appropriate. In contrast, 10 respondents determined this as an ineffective strategy:

“*A lot of patients who have had treatment suspended due to stable disease now (6 weeks later) seem to be showing progression. Difficult to know if this [suspending treatment] was the right thing to do.”*

*“Postpone the benign treatment. This will result in a long queue and the size [of the] benign lesion is getting bigger which [can] cause many symptoms”*

### Distancing in the workplace

30 respondents believed distancing in the workplace to be ineffective, highlighting barriers such as limited capacity for blood testing and other activities. Lack of space in the hospital was seen as a problem in being able to maintain safe distances. There was additional concern over patients being disallowed companions during treatment.

“*Insistence of staff staying 1.5m apart, this has not been physically possible with the number of staff we have our small area*”.

## Discussion

The survey results have shown some universal strategies that have been implemented and perceived as effective by clinicians from around the world, with the most effective being the utilisation of telehealth and treatment closer to home. Treatment closer to home for cancer care has been understood to benefit many patients and is a model of care implemented where distances to hospital are barriers. Interestingly, despite the model having benefits for patients and providers of health care, it was seldom utilised prior to the pandemic.^11^ The rapid implementation of technology and pathways including home care have enabled patients to both continue treatment through the pandemicSome strategies such as switching to oral treatments and treatment breaks are less understood. It is not clear if these strategies will have long term implications on a patients’ cancer. Some oral treatments are equivalent to the intravenous ones, but others are newly licensed and their place in the treatment pathway is yet to be determined.^12^ Using routinely collected national prescribing data from this period could guide future pathway decisions for patients. Our findings are supported by a recently published international survey of oncology clinicians. This survey reported a 7.7-fold increase in the proportion of consultations using telehealth, however 25% of clinicians reported concerns that increased telehealth could lead to a worsened patient survival because of less frequent physical assessments.^13^

Concerns about the psychological impact of new models of care should also be taken into consideration.

We undertook this work in order to disseminate strategies implemented around the world and help others. Since our survey, international societies such as The American Society of Clinical Oncology (ASCO) and The European Society of Medical Oncology (ESMO)have provided consensus guidance in changing practice^14^ that are concordant with our findings; however, our work is still valuable to share, highlighting challenges that are faced in the health service. Distancing in the workplace impacts on biopsies, blood tests and chemotherapy reconstitution; all these areas will need to be addressed in order to continue providing a chemotherapy service which not only protects the workforce but also has the lowest impact on patients such as increased patient waiting times. Our work has been important in highlighting some of the challenges faced at the beginning of the pandemic and challenges that will continue. Although it is clear that COVID-19 related outcomes are poor in patients with cancer^15–17^ the impact of anti-cancer therapy on those outcomes, remains contentious. The risk chemotherapy poses on death from COVID-19 has been noted by authors to be low. In the United States population this was calculated as HR, 1.10; 95% CI, 0.73 to 1.60 and similarly in the United Kingdom a HR of 1·18 95% CI, 0·81–1·72. Models to calculate this risk were, however, based on data collected at the time of our survey and may not represent the whole chemotherapy patient population and therefore should be used with caution.^1,15^

We were fortunate, despite the busy timing, to receive 129 responses and attain the sample size planned, reaching saturation of themes; but we would have liked more respondents from Low- and Middle-Income Countries (LMICs) to understand challenges that were faced in these settings. Furthermore, we only gained one respondent in the US and only one chemotherapy nurse answered our survey. Our survey does not put the responses into the context of case numbers at the time of the survey, however the survey by Chazan *et al* reports that 88.8% of respondents altered cancer management or the delivery of cancer services, with similar rates across Europa, Asia and Australia/New Zealand, despite disparities in COVID-19 case numbers.^13^

The survey was open for a short duration, only capturing a snapshot of experiences and strategies implemented during the pandemic. It is possible that there is respondent bias in the sense that respondents working in hospitals implementing strategies aimed at patient prioritisation were more willing to take part in the survey.

## Conclusions

The pandemic has forced those working within chemotherapy to adopt new ways of working to reduce health interactions. There will be many areas of research following this period, including understanding patients’ perceptions of risks to treatment, utilisation of oral treatments and the impact of treatment breaks on cancer outcomes. We believe that many models adopted such as telehealth will continue, giving patients’ flexibility in how they receive their care in future, however the impact on patient outcome and the patient perspective on these new models of care needs to be further studied.

## Supplemental Material

sj-pdf-1-opp-10.1177_10781552211015767 - Supplemental material for Global changes to the chemotherapy service during the covid-19 pandemic Click here for additional data file. Supplemental material, sj-pdf-1-opp-10.1177_10781552211015767 for Global changes to the chemotherapy service during the covid-19 pandemic by Man-Chie Chow, Pinkie Chambers, Georgina Singleton, Jignesh Patel, Silvie Cooper, Charlotte Mythen, Elysse Bautista-González, Georgia Chisnall, Nehla Djellouli, Benjamin Thwaites, Ian CK Wong and Cecilia Vindrola-Padros in Journal of Oncology Pharmacy Practice

sj-pdf-2-opp-10.1177_10781552211015767 - Supplemental material for Global changes to the chemotherapy service during the covid-19 pandemic Click here for additional data file. Supplemental material, sj-pdf-2-opp-10.1177_10781552211015767 for Global changes to the chemotherapy service during the covid-19 pandemic by Man-Chie Chow, Pinkie Chambers, Georgina Singleton, Jignesh Patel, Silvie Cooper, Charlotte Mythen, Elysse Bautista-González, Georgia Chisnall, Nehla Djellouli, Benjamin Thwaites, Ian CK Wong and Cecilia Vindrola-Padros in Journal of Oncology Pharmacy Practice
