# Supplementary material for: Global changes to the chemotherapy service during the covid-19 pandemic
Source: J Oncol Pharm Pract. 2021 May 13;27(5):1073–9. doi: 10.1177/10781552211015767 (PMC8367193; doi:10.1177/10781552211015767)
Supplement: sj-pdf-1-opp-10.1177_10781552211015767 - Supplemental material for Global changes to the chemotherapy service during the covid-19 pandemic [file sj-pdf-1-opp-10.1177_10781552211015767.pdf]

Acknowledgements
We would like to thank the following professional societies for their support in dissemination of the survey; The British Oncology Pharmacists Association; The Royal College of Radiologists; The International Society of Oncology Pharmacy Practitioners; The UK Oncology Nursing Society; The British Urology Group. Additionally, we would like to thank the following clinicians that contributed to the survey development; Dr Martin D Forster; Jennifer Jupp and Dr Gabriel Brooks.

Declarations

Funding
No funding was received for conducting this study

Conflicts of Interest/Competing interests
Authors GS, JP, SC,CV, CM, BT, EB, GC and ND have no conflicts of interest. The following authors have disclosures: Ms Chambers reports a research grants from Bristol Myers Squibb not during the conduct of this study,Ms Chow reports educational grants from Mundipharma, Sanofi Genzyme, Janssen, Takeda and Celgene not during the conduct of this study; Professor Wong reports research grants from Bristol Myers Squibb not during the conduct of this study. Author PC is a Doctoral Research Fellow funded by the NIHR Academy (Project reference DRF 2017-10-016) The views expressed are those of the authors and are not necessarily those of the NHS, the NIHR or the Department of Health and Social Care. Man-Chie Chow is a funded by The Royal Marsden Partners, PAN London Fellowship Scheme.

Ethics Approval
The study, including the questionnaire, was approved by the University College London Research Ethics Committee (6862/005).

Consent to Participate: N/A

Consent for Publication: N/A

Availability of data
Authors have full control over data extracted and agree for this to be reviewed on request.

Code Availability: N/A

Authors Contributions
All authors were involved in the conception and design of the work; authors GS JP, PC, ND, IW EB and CV developed the data collection tool ; authors SC, CM, CV, CS and MC and PC were involved in data analysis. All authors contributed to the drafting and revisions of the manuscript; all authors have finally approved the manuscript and agree to be accountable for all aspects of the work.
