# Supplementary material for: Global changes to the chemotherapy service during the covid-19 pandemic
Source: J Oncol Pharm Pract. 2021 May 13;27(5):1073–9. doi: 10.1177/10781552211015767 (PMC8367193; doi:10.1177/10781552211015767)
Supplement: sj-pdf-2-opp-10.1177_10781552211015767 - Supplemental material for Global changes to the chemotherapy service during the covid-19 pandemic [file sj-pdf-2-opp-10.1177_10781552211015767.pdf]

COVID-19 and Systemic Anti-cancer Therapy Survey

Preface

In light of the COVID-19 pandemic, there have been mitigation strategies implemented, either nationally or within local hospitals, for the management of patients that are at high risk such as those receiving cancer chemotherapy. We developed a survey to capture the views of healthcare professionals currently delivering cancer care.

The survey is developed by an international interdisciplinary team with researchers from the Rapid Research, Evaluation and Appraisal Lab (RREAL), University College London and University College London NHS Foundation Trust. To obtain more information about this collaboration please contact Dr Pinkie Chambers (p.chambers@ucl.ac.uk).

Research findings will enable a better understanding of the current global measures. This information will also be important for future virus outbreaks should they ever occur.

1. Who has given ethical approval for this study?
This study was approved by the University College London (UCL) Research Ethics Committee (REC).

2. Why have I been asked to take part?
We wish to capture a wide range of views from the people in charge of designing, implementing and interacting with the delivery of cancer care in the current pandemic climate. We believe you have a valuable perspective.

3. What does taking part involve?
The survey will include questions about your current role and your experience of current cancer care policy and implementation.

4. Do I have to take part?
No, it is up to you to decide whether or not to take part. If you decide to take part and click on the link to complete the survey, this will be taken as consent.

5. Is what I say confidential?
Yes, we will not inform anyone outside the research team that you have participated in the study. All information will be stored securely and will only be accessed by members of the research team. Your data will be archived securely for 20 years after the study's completion, before its eventual destruction.

6. What if I change my mind?
You are free to withdraw from the survey at any time. Even if you start the survey, you can stop it at any point if you wish to.

7. What are the risks of taking part?
Helping us with this study will take up a little of your time. If you feel uncomfortable discussing any aspect of this study, you can withdraw from the survey at any time. You can also contact the study team to discuss any concerns you have before or after agreeing to take part.

8. What are the benefits of taking part?
There may be limited personal benefits emerging from the study, but the study aims to disseminate how countries around the world are managing patients that would normally have received systemic anti-cancer therapy. This will be important for future virus outbreaks if they should ever occur. The final results from the study will be shared across relevant networks and will be made available on the RREAL website.

9. How will information be stored?
UCL is the sponsor for this study. We will be using information from you in order to undertake this study and will act as the data controller for this study. This means that we are responsible for looking after your information and using it properly. UCL will keep the information collected for this study for 20 years after the study has finished. Your rights to access, change or move your information are limited, as we need to manage this information in specific ways in order for research to be reliable and accurate. If you withdraw from the study, we will keep the information that we have already obtained. To safeguard your rights, we will use the minimum personally identifiable information possible. You can find out more about how we use the information collected for this study by contacting the research team using the contact details listed below.

10. What will happen to the results of the research study?
The final results from the study will be shared across relevant networks and will be made available on the RREAL website. We will aim to publish our findings in scientific journals and present them at national and international scientific meetings and conferences.

11. What happens if something goes wrong?
If you wish to complain or have any concerns about any aspect of the way you have been approached or treated through your participation in the research, you may wish to contact the researchers (details below).

12. Local Data Protection Privacy Notice
The controller for this project will be University College London (UCL). The UCL Data Protection Officer provides oversight of UCL activities involving the processing of personal data, and can be contacted at data-protection@ucl.ac.uk. This 'local' privacy notice sets out the information that applies to this particular study. Further information on how UCL uses participant information can be found in our 'general' privacy notice.

The information that is required to be provided to participants under data protection legislation (GDPR and DPA 2018) is provided across both the 'local' and 'general' privacy notices. We will be collecting data on your country, type of institution and professional role. The lawful basis that will be used to process your personal data are: 'Public task' for personal data. Your personal data will be processed so long as it is required for the research project. If we are able to anonymise or pseudonymise the personal data you provide we will undertake this, and will endeavour to minimise the processing of personal data wherever possible.

If you are concerned about how your personal data is being processed, or if you would like to contact us about your rights, please contact UCL in the first instance at data-protection@ucl.ac.uk.

Researchers
Principal Investigator: Dr Pinkie Chambers (p.chambers@ucl.ac.uk)
Co-Investigator: Professor Ian Wong (i.wong@ucl.ac.uk)
Co-Investigator: Dr Cecilia Vindrola (c.vindrola@ucl.ac.uk)
If you have any questions or complaints, you can also contact the UCL Research Ethics Commitee (ethics@ucl.ac.uk)

Thank you for taking the time to read this information and for considering helping with our study. Participation is voluntary, clicking 'start' will be taken as consent.

COVID-19 and Systemic Anti-cancer Therapy Survey

A. The following questions are about you and your workplace

i. Which country do you work in?
(free text)

ii. What type of institution do you work in?
❒ Public
❒ Private
❒ Hybrid (public & private)
❒ Other, please specify (free text)

iii. Does your institution **only** manage cancer patients?
❒ Yes
❒ No

iv. What type of healthcare professional are you?
❒ Pharmacist
❒ Doctor
❒ Clinical nurse specialist
❒ Chemotherapy nurse
❒ Other, please specify (free text)

B. [Delivery of systemic anti-cancer therapies]

v. What is the current status of the delivery of systemic anti-cancer therapies at your institution?
❒ All treatments are continuing
❒ Some (but not all) treatments are continuing
❒ All treatments are cancelled or postponed
❒ I don't know

if b or c (branching to incl vi) -->

vi. Why have treatments been cancelled or postponed? (please select all that apply)
❒ Revised individual risk-benefit balance (in view of COVID-19)
❒ Lack of staff owing to redeployment to other clinical areas
❒ Lack of staff owing to staff illness or isolation
❒ Lack of bed capacity and/or equipment
❒ Insufficient resources to shield cancer patients from COVID-19
❒ Cancer care has moved to another site
❒ Other, please specify (free text)

C. The following questions are about the prioritisation of systemic anti-cancer therapies during the COVID-19 pandemic

vii. Are you aware of any ***national guidelines*** in your country offering recommendations on how to ***prioritise*** systemic anti-cancer therapies?
❒ Yes

- ❒ No

viii. Are there any ***local (institutional) policies*** for the ***prioritisation*** of systemic anti-cancer therapies if required?

- ❒ Yes
- ❒ No

ix. Has there been a need to ***implement prioritisation strategies*** at your institution?

- ❒ Yes
- ❒ No

If yes (branching to include x and xi) -->

x. Based on your experience so far, in the event of a future pandemic requiring prioritisation of systemic anti-cancer therapies, which of your prioritisation strategies would you ***re-employ*** and why?
(Please provide as much detail as possible of the strategy, including any prioritisation categories and thresholds e.g. % chance of cure)
(free text)

xi. Based on your experience so far, in the event of a future pandemic requiring prioritisation of systemic anti-cancer therapies, which of your prioritisation strategies would you ***change*** and why?
(Please provide as much detail as possible of the strategy you would change and your reasons for change)
(free text)

D. <u>The following questions are about the strategies in place to protect patients from COVID-19.</u>

xii. Are you aware of any ***national guidelines*** in your country offering recommendations for the ***protection of patients*** undergoing systemic anti-cancer therapies from COVID-19?

- ❒ Yes
- ❒ No

xiii. Are there any ***local (institutional) policies*** for the ***protection of patients*** undergoing systemic anti-cancer therapies from COVID-19?

- ❒ Yes
- ❒ No

xiv. Has your institution ***implemented*** any of the following strategies to ***minimise face-to-face contact*** between patients undergoing systemic anti-cancer therapy and health care workers? (Please select all that apply)

- ❒ Telephone/video remote clinics
- ❒ Reduced frequency of non-essential clinic follow-up
- ❒ Reduced frequency of non-essential follow-up investigations
- ❒ Use of personal protective equipment (PPE) for all staff interacting with patients
- ❒ Other methods to minimise face to face contact - please specify (free text)

xv. Has your institution ***implemented*** any of the following ***social-distancing*** measures? (Please select all that apply)

- ❑ No family or friends to accompany patient to healthcare appointments
- ❑ Minimising time in clinic / chemotherapy unit (e.g text message when clinician available for appointment)

- ❑ Closure of waiting rooms / seating areas / cafes
- ❑ Changes in layout of the chemotherapy unit to increase distancing
- ❑ Using local rather than central services for investigations
- ❑ Home treatments
- ❑ Home delivery of medications
- ❑ Other social distancing measures - please specify (free text)

xvi. Has your institution ***implemented*** any of the following ***changes to systemic anti-cancer therapy regimens***? (please select all that apply)

- ❑ Less immunosuppressive regimens
- ❑ Reduced frequency or duration of treatments
- ❑ Change to the route of administration (e.g. IV to PO)
- ❑ Increasing primary prophylaxis (e.g. antiemetics, antibiotics)
- ❑ Changes to the timing of therapy (e.g. treatment breaks)
- ❑ Other changes to systemic anti-cancer therapy regimens - please specify (free text)

xvii. Is your institution ***screening / testing*** all asymptomatic patients undergoing systemic anti-cancer therapies for COVID-19?

- ❑ Yes
- ❑ No

xviii. Please specify ***any other strategies*** employed by your institution to help protect patients from COVID-19 (free text)

xix. Of all the ***implemented*** strategies to protect patients from COVID-19 at your institution, which do you believe has been the ***MOST effective*** and why? (free text)

xx. Of all the ***implemented*** strategies to protect patients from COVID-19 at your institution, which do you believe has been the ***LEAST effective*** and why? (free text)

xxi. Please include any other information that you think is important for us to know. (free text)

xxii. How did you find out about this survey?

- ❑ organisation A
- ❑ organisation B
- ❑ organisation C
- ❑ organisation D
- ❑ organisation E
- ❑ personal contact
- ❑ other, please specify (free text)
